# Culturally Adapting a Video-Based Self-acupressure Intervention to Manage Symptoms for Black and Latina Breast Cancer Survivors

**DOI:** 10.1007/s13187-025-02684-1

**Published:** 2025-07-12

**Authors:** Katarina E. AuBuchon, Amrita Bonthu, Lourdes Inbar-Albo, Thelma D. Jones, Jacqueline Beale, Claudia Campos-Galván, Laura A. Logie, Carla Arieta, Geng-Hao Liu, Alejandra Hurtado-de-Mendoza, Suzanne C. Danhauer, Kristi D. Graves, Judy Huei-yu Wang

**Affiliations:** 1 Department of Oncology, Georgetown University, Washington, DC USA; 2 Department of Medicine, Case Western Reserve University School of Medicine, Cleveland, OH USA; 3 Thelma D. Jones Breast Cancer Fund, Washington, DC USA; 4 C2JB Consulting, Glenn Dale, MD USA; 5 The George Washington University Cancer Center, Washington, DC USA; 6 Nueva Vida, Inc, Alexandria, VA USA; 7 Inspire Integrative Medicine, Arnold, MD USA; 8 School of Traditional Chinese Medicine, Graduate Institute of Traditional Chinese Medicine, College of Medicine, Chang Gung University, Taoyuan, Taiwan; 9 Division of Acupuncture and Moxibustion, Center for Traditional Chinese Medicine, Linkou Chang Gung Memorial Hospital, Taoyuan, Taiwan; 10 Department of Social Sciences & Health Policy, Division of Public Health Sciences, Wake Forest University School of Medicine, Winston-Salem, NC USA

**Keywords:** Breast cancer, Acupressure, Cultural adaptation, Survivorship

## Abstract

Black and Latina breast cancer survivors (BCS) experience greater physical functioning decline and symptom burden than non-Hispanic White BCS, partially due to systemic racism and socioeconomic barriers. Self-administered acupressure has shown promising effects for reducing cancer-related symptoms and is a low-cost, integrative approach to managing symptoms. However, self-acupressure has not been tested in Black and Latina BCS. This study aimed to culturally adapt an existing self-acupressure video intervention and assess Black and Latina BCS’s feedback on the acceptability of the adapted video. Following learner verification and revision methods, we collaborated with community organizations and partners for cultural and linguistic adaptation of the existing video. We conducted semi-structured interviews with four community partners, two oncology providers, and six Black and Latina BCS to gather feedback on content comprehension, engagement, cultural acceptability, and satisfaction with the intervention. We made iterative revisions to the video and then conducted usability testing with the six BCS. We produced an 11-min self-acupressure video following participant feedback, including a need for clear differentiation between acupressure and acupuncture, simplified explanations of physiological effects of self-acupressure, increased culturally relevant presentation by including Black and Latina BCS performing acupressure demonstrations, testimonial segments, and lay language explanations. In usability testing, the six BCS rated the intervention highly. Black and Latina BCS were highly satisfied with the culturally adapted self-acupressure video and expressed strong interest in performing self-acupressure. Future studies will evaluate the intervention’s effects on symptom management and quality of life in these underrepresented populations.

**Supplementary Information:**

The online version contains supplementary material available at 10.1007/s13187-025-02684-1.

## Introduction

After completing their first course of cancer treatment, cancer survivors experience worse functioning compared to the general population [1], which contributes to functional decline. Due to historical and structural racism that impacts many factors (e.g., care access) [2, 3], Black and Latina BCS consistently demonstrate worse physical functioning and symptoms than non-Hispanic White BCS [4, 5]. Thus, it is imperative to help Black and Latina BCS manage symptoms and promote functioning. Patients with cancer report growing interest in non-pharmacological, low-cost, and accessible approaches to symptom management, and half (51%) report using integrative medicine to manage symptoms [6]. Despite Black and Latina patients’ interest in integrative medicine [7, 8], they are vastly underrepresented in integrative medicine research.

In prior studies, Black cancer survivors gravitated toward self-directed physical therapies to manage cancer symptoms [9], and Black and Latina cancer survivors experience more barriers to intervention engagement that require frequent visits with instructors and cost [10]. These barriers likely reflect socioeconomic concerns, which are more likely to occur among Black and Latina BCS due to structural racism and immigration impacting employment type, job flexibility, and income equality [11, 12].

Self-administered acupressure (*self-acupressure*), rooted in Traditional Chinese Medicine (TCM), is an integrative intervention for alleviating cancer-related symptoms that is a low-cost, low-intensity, and safe symptom management method [13]. In self-acupressure, people use their thumbs or fingers to stimulate “acupoints” on the body, which maintains internal organ functions by stimulating meridian pathways to circulate blood and *qi*, a person’s vital force/energy [14]. From the Western biomedical perspective, acupressure applies Pascal’s principle–pressure applied to an enclosed fluid transmits undiminished force [14]. Acupressure is theorized to release anti-inflammatory hormones like glucocorticoids that modulate the immune response and the sympathetic nervous system to reduce inflammation and improve energy [15, 16]. In randomized controlled trials (RCTs), self-acupressure is efficacious in reducing cancer-related symptoms [13, 17, 18]. However, none of these studies evaluated self-acupressure among Black or Latina BCS, and if they included Black or Latina BCS, they had limited representation. Self-acupressure is self-directed, requires no materials, and may be suitable for symptom management among Black and Latina BCS.

The purpose of the present study was to adapt an existing self-acupressure video intervention and conduct usability testing of the adapted video. The existing video [19] was originally designed for Chinese- and English-speaking patients with solid tumors; we sought to culturally and linguistically adapt the video to suit Black and Latina BCS. Cultural adaptation involves enhancement of existing interventions by altering the content, language, delivery method, or other elements for specific populations [20]. We followed learner verification and revision [21], an approach that engages patients and stakeholders to develop, revise, and refine the materials to ensure that the educational messages in the materials are culturally and linguistically appropriate for our target audience. Following iterative revisions, we conducted usability testing to assess Black and Latina BCS’ satisfaction with and acceptability of the adapted self-acupressure intervention.

## Methods

The protocol was approved by the Georgetown-MedStar Institutional Review Board.

### Study Setting and Population

We used a community-based participatory research approach, working with community organizations—Nueva Vida, Inc. and the Thelma D. Jones Breast Cancer Fund—that serve Black and Latina BCS in Washington, DC, the largest and second-largest historically marginalized racial and ethnic groups in DC, respectively [22]. Washington, DC is a city emblematic of extreme inequity; Black and Latina DC residents experience worse breast cancer outcomes, including higher mortality relative to White DC residents [23].

Community organizations invited community partners to participate in the adaptation of the self-acupressure intervention video. The two organizations have long served Black and Latina BCS in the metropolitan Washington, DC area, respectively, and collaborated with the research team on various projects. Community partners included community leaders and patient advocates, of whom two were also BCS. These partners referred Black and Latina BCS to participate in this study and were involved in intervention adaptation. We recruited six BCS (50% Black and 50% Latina) who were diagnosed with breast cancer and at least 1 year post-treatment), and 2 breast oncologists and 4 community partners (total *N* = 12) who also identified as Black or Latina/o to participate in semi-structured qualitative interviews (see Table 1 for demographics). The six BCS were then subsequently invited to participate in usability testing of the adapted intervention video.

**Table 1:** Participant demographics

	Breast cancer survivors 6 (50%)	Community partners and providers 6 (50%)	Total 12 (100%)
Gender			
Man	0 (0%)	1 (17%)	1 (8%)
Woman	5 (83%)	5 (83%)	10 (84%)
Racial group			
Black/African American	2 (33%)	2 (33%)	4 (33%)
Hispanic/Latina	3 (50%)	4 (67%)	7 (59%)
Born in the US?			
Yes	1 (20%)	2 (33%)	3 (27%)
No	2 (40%)	2 (33%)	4 (36%)
Education level			
High school or below	2 (33%)	-	2 (17%)
Some college and above	3 (50%)	-	9 (75%)
Employment status			
Employed	4 (66%)	5 (83%)	9 (75%)
Unemployed or retired	1 (17%)	1 (17%)	3 (27%)
Health insurance status			
Insured	4 (66%)	6 (100%)	10 (91%)
Uninsured	1 (17%)	-	1 (9%)
Age			
< 45	1 (17%)	1 (17%)	2 (17%)
45–54	2 (33%)	2 (33%)	4 (33%)
55–64	1 (17%)	1 (17%)	2 (17%)
65–74	1 (17%)	2 (33%)	3 (25%)

*Note*. One BCS declined to complete the demographic survey; thus, percentages do not sum to 100%. Five participants declined to respond about being born in the USA

### Cultural Adaptation of the Self-acupressure Video Intervention

**Fig. 1:**
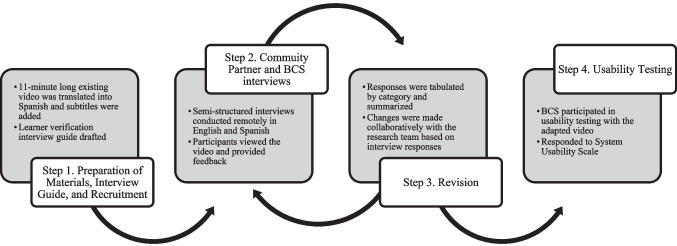
Learner verification and revision. *Note.* Cultural adaptation (steps 1–3) and usability testing (step 4) processes, following learner verification and revision

We followed learner verification and revision [21] to adapt an acupressure educational intervention video for Black and Latina BCS (Fig. 1) in three steps: (1) preparation of materials including initial changes and developing an interview guide; (2) participant interviews; and (3) analysis of data from participant interviews and iterative revision of materials. Lastly, we conducted usability testing with the adapted materials. At each phase, we assessed the five key elements of learner verification and revision: content comprehension (understanding materials), attraction (engagement and appeal of materials), self-efficacy (confidence in ability to perform the intervention), cultural acceptability (culturally appealing and agreeable), and persuasion (intervention is compelling) [21].

#### Step 1. Preparation of Materials

The study team reviewed a pre-existing video in Chinese and English languages from previous work [19]. The video explained the mechanisms and delivery of self-acupressure in three sections (see Table S1 in supplementary materials): a brief introduction of acupressure theory, location of the three key energy acupoints (LI4, ST36, SP6; Fig. S1 in supplementary materials) on each side of the body, and directions on how to press the acupoints correctly. In the video, a female acupuncturist (CA, a co-author) introduced acupressure in English in the same theory as acupuncture, noting that these two techniques use different methods of stimulation. The narration continued in English for the physician (CA, co-author) who demonstrated how to press on each acupoint, explained sensations when an acupoint is correctly pressed (e.g., tingling, warm, and/or aching, known as “Deqi”), and when to stop performing. A Spanish script was provided for Spanish-speaking participants.

We crafted a semi-structured interview guide, revised with input from community partners, focusing on the aforementioned five elements of learner verification and revision: (1) content comprehension (e.g., “Do you think you could identify the acupoints after this video?”); (2) attraction (e.g., “What were your thoughts on the display and pictures?”); (3) intervention self-efficacy (e.g., “[Based off of the video] will women do the acupressure each day?”); (4) satisfaction and persuasion (e.g., “Would you watch this video again to learn self-acupressure?”); and (5) cultural acceptability (e.g., “Do you have any suggestions for altering it to make it more accessible to Latina and Black women?”). This interview guide was drafted in English by English-speaking and bilingual (English–Spanish) study team members. After the guide was drafted, one of the native Spanish-speaking study team members translated the guide into Spanish, and then another native Spanish-speaking team member reviewed it to revise or confirm the translation. Questions on specific wording choices were discussed with the team.

#### Step 2. Phased Participant Interviews

We conducted semi-structured qualitative interviews with each of the 12 participants to understand their feedback on the existing acupressure intervention. We adopted a phased approach to the interviews to allow for iterative revision and refinement of the materials for the production of the acupressure video and intervention. First, we interviewed two oncologists (English) and four community partners (English or Spanish) to obtain their feedback on the existing English-language acupressure educational video. Second, we conducted interviews with the six Black and Latina BCS in English or Spanish, respectively. Trained bilingual research assistants (RAs) conducted individual interviews remotely (e.g., via Zoom), except one conducted in person. Interviewers provided each participant with an overview of the study purpose prior to documenting informed consent. Before the interviews, we reiterated that we were interested in participants’ opinions on the materials and that there were no right or wrong answers. Study team members then used the semi-structured guide to begin the interviews. Each interview lasted 30–45 min and was recorded. Spanish interviews were translated into English for analysis and transcribed verbatim, and English interviews were transcribed verbatim for analysis.

#### Step 3. Analysis of Interview Data and Iterative Video Adaptation

The team analyzed the qualitative data following the same five elements of learner verification [21]. The trained RAs reviewed participants’ responses to questions regarding each element and then tabulated them under each learner verification element for the research team to discuss. Following a phased approach, we iteratively revised the video script and content based on feedback from the oncologists and community partners prior to interviews with the six Black and Latina BCS. Based on BCS feedback, we further revised the video for usability testing.

#### Step 4. Usability Testing

Last, we conducted usability testing with the same six BCS who participated in the step 2 interviews. By conducting usability testing with the same BCS, we could ensure we accurately interpreted their feedback and applied the correct adaptations to the video. During usability testing, participants first viewed the 11-min video via a URL and then participated in another structured interview, using similar questions, to address the same five elements of interest for the video. Participants also provided quantitative usability data by responding to the System Usability Scale (SUS) from 0 (strongly disagree) to 4 (strongly agree) [24]. The study team summarized qualitative and quantitative responses to establish acceptability of the adapted self-acupressure video intervention.

## Results

### Participant Interview Results

Qualitative interview results are in Table 2. In general, participants agreed that the length, information included, and pace of the video were ideal. They reported that the information was persuasive, but additional changes could aid in comprehension of materials. BCS described a need for better explanation about the distinction between acu*puncture* vs. acu*pressure*. All participants appreciated that the video provided easy-to-follow instructions on finding each acupoint using easily identifiable landmarks on the body. However, more details about the symptom management benefits were requested, including adding images. Continued adaptation of terms to lay language was needed (e.g., “medial”) and terms for acupoints that were user-friendly (e.g., “L14”).

**Table 2:** Results from learner verification and revision semi-structured interviews

Feedback from step 2 interviews									
Providers			Community partners			Patients			Summary
Content comprehension									
“I think it’s very clear. There are things patients will be thinking about and not paying attention to it so maybe say both hands instead of bilateral. At the beginning of the video, she said acupoints, instead maybe she should say we will explain later so patients are not confused or asking themselves what are acupoints? Instead of listening.” – Latina Provider			“If this is acupressure, why are we starting with acupuncture? Immediately people are going to dismiss since you started with acupuncture. Even though you explain there is a difference between the two, people will correlate the two which may make them apprehensive.” – Black woman community partner “…have a graphic that shows how acupressure can help you. She talks about it in the introduction but sometimes a voiceover is good. Some graphic with reducing nausea, pain, etc. that may be a good thing.” – Latina community partner			“I was not aware this is self-acupressure since I have had acupressure before, but the self should be announced. I would like to see it more exciting and inviting. […]There should also be some type of graphic to emphasize acupressure versus acupuncture.” – Black BCS “And the L14 point does not mean anything to anyone. Maybe come up with an acronym to remember the 3 points by the hand, knee, calf. The average person will not care about the specific term like L14.” – Black BCS			• Need to explain the difference between acupressure and acupuncture and massage • Need to explain why some acupoints are helpful for certain bodily functions • Need clear explanations of location of the different acupoints • Need to describe physiology and procedures in lay terms
Attraction									
“I think [the format, images, and layout] are nice, easy to follow. I think it was a good balance.” – Latina provider		“Just like you have a graphic with the four steps (pointing, pressing, kneading, pushing), you might want to have a graphic that shows how acupressure can help you. She talks about it in the introduction but sometimes a voiceover is good. Some graphic with reducing nausea, pain, etc. that may be a good thing.” – Latina community partner			“The speakers need to sound excited; they didn’t sound excited.” – Black BCS “…it [would] be great if a cancer survivor can share their experience. I suffer from cramps after chemo sections. So, it [would] be good to hear from a cancer survivor about the benefits.” – Latina BCS			• Include real people in demonstrations • Describe acupressure benefits relevant for the population	
Self-efficacy									
“Maybe having the patient doing [acupressure] as she explains. […] Something short, around 20 s like this helped my fatigue.” – Latina Provider “Emphasize this can be done at home, so patients don’t have to go to the hospital.”	“One woman sharing the benefits with [the] other women and having something simple for people to watch that shows the benefit.” – Latina community partner “One thing might want to mention, not only can they do it in a relaxing position, but they can do it anywhere. They do not have to be in the house, they can be in the park, outside or even in their car. They can practice this anywhere.” – Black woman community partner			“There was not very much excitement about it. Put emphasis on what acupuncture is and this is acupressure, and you can do it yourself to get people excited about it. […] [M]ention symptoms of people going through treatment and what they can do to help with them so It sounds like it really is for them.” – Black BCS			• Include testimonials from other breast cancer survivors (BCS) to enhance credibility • Emphasize the simplicity and effectiveness of self-care through self-acupressure • Highlight personal success stories to reinforce the ease and benefits of the practice		
Cultural acceptability									
“[Have] Black or Latina patients providing testimonials. We underestimate the power of someone that look likes them saying that it works.” – Latina Provider	“If this study is for Black, African Americans, and Latinos then you should probably have some pictures of people depicting those demographics.” – Black woman community partner “It is important to create a video in Spanish using the word acupresión.” – Latina community partner			“If [you’re] trying to use it for both Black and Latina, might want to use a Black speaker as well.” – Black BCS “It [would] be good to translate this video in Spanish” – Latina BCS			• Include diverse representation in the videos • Translate the video into Spanish		
Persuasion									
“I think the video is clear. Great length, not too long. The diagrams and dots were very helpful [to teaching acupressure].” – Latino Provider	“I loved the video. Anybody can learn how to do it which is what I was focusing on, like how simple is it for people to learn how to do self-acupressure. And I think anybody can do this.” – Black women community partner			“That was good, not too long, or short.” – Black BCS “All the graphics and explanations given by the Doctor were self-explanatory and detailed.” – Latina BCS			• The length and pace of the video were good • Positive feedback on the instruction and layout of the video		

BCS expressed that the physician was speaking too fast and her voice was monotone in the video. Participants repeatedly asked for more “excitement.” The original video included “serene” images and static images, whereas the Latina and Black BCS requested more movement, varied images, and colorful graphics. Community partners and BCS indicated that testimonials and the inclusion of real patient demonstrations would increase video engagement and appeal. Participants also expressed that testimonials would increase self-efficacy, enhance the video’s credibility, and increase viewers’ motivation to practice. BCS commented that patient excitement in the video could make people feel like acupressure is “really for them.” An additional theme was to include diverse representation in the videos; the original video included people with lighter skin, which made the video less relatable to participants. Similarly, having the video fully translated into Spanish was a key improvement for Latina BCS.

### Cultural Adaptation of the Self-acupressure Video

To culturally adapt the video, we made adaptations based on the qualitative interview results from Black and Latina BCS and Black and Latina/o community members and providers in the content of the video and surface-structure (i.e., appearance) of the video. To enhance comprehension, the physician used simpler terminology and lay terms to explain differences in acupressure and acupuncture, addressing physical risks and how acupressure influences health, and TCM concepts (e.g., Qi) in the adapted video. These were areas identified by our interview participants as key adaptations. Additionally, we addressed health literacy concerns, ensuring the video used clear, easy-to-understand language. For engagement and appeal, the physician’s tone was altered to increase inflections and positive tone, and she spoke at a slower pace with clearer pronunciation. The adapted videos included colorful graphics and varied images to promote engagement and appeal.

The adapted video emphasized the safety and accessibility of self-acupressure by incorporating BCS practicing self-acupressure alone. Following participant and stakeholder feedback, we also included images related to the practice of acupressure with people who had both short and mid-length fingernails, and noted that nails that were too long may reduce the ability to provide the correct amount of finger pressure. To address cultural acceptability of the video, we included testimonials from Latina and Black BCS who shared their personal experiences with self-acupressure. The addition of Black and Latina women performing acupressure also was a specifically requested adaptation from the interview participants. Additionally, the video was fully translated into Spanish for Latina BCS.

### Usability Testing of the Culturally Adapted Self-acupressure Video

Usability testing indicated that the adapted video was well-received by all six BCS, who found it easier to understand and more visually appealing than the previous video. On average, participants rated usability highly (*M* = 3.96, SD = 0.2 on 1–5 scale). Participants expressed that the adapted video was more enthusiastic and colorful. The clear explanations, effective demonstrations, and helpful testimonials made the content relatable and motivating to perform acupressure. Qualitative feedback about the testimonials was especially positive, highlighting their value as a key addition to the video. After viewing the video, participants endorsed that practicing self-acupressure could bring various benefits, such as stress relief and symptom alleviation, and they were excited to try it themselves and share it with others. Following usability testing, minor changes were made, such as fixing grammatical/spelling errors. Additionally, Spanish-speaking BCS expressed having voiceovers (vs. subtitles) in Spanish instead of English in the testimonials would aid comprehension.

## Discussion

The current integrative medicine literature has a dearth of representation of Black and Latina people in study development and testing, particularly in cancer survivorship. The current project filled this gap by working with community partners and BCS to create a culturally relevant adaptation of a previously designed acupressure intervention. Our qualitative findings confirmed the effects of community engagement and cultural relevance for increasing Black and Latina patients’ high interest in using integrative approaches to cancer symptom management ^7,8^. The hypothesized physiological impacts of acupressure on symptom management are applicable for all people, regardless of ethnic background, and our results illustrate the acceptability and feasibility of integrative approaches with Black and Latina BCS.

Our study identified that adapting the intervention was helpful for increasing interest and motivation in the intervention. Adapting the intervention using learner verification and revision is an effective method of making an intervention engaging and suitable to patients. Our approach addressed both surface-level (e.g., colourful pictures/graphics) and deep-level (e.g., testimonials) changes to encourage interest in our target population. Ample research supports that cultural adapting of behavioral interventions is key to success [25]; however, such adaptation has not occurred with acupressure approaches. Especially given Black and Latina cancer survivors’ interests in integrative medicine, researchers should incorporate culturally specific feedback early to ensure intervention reach to diverse audiences. Our study supports that integrative medicine interventions can be adapted for patients from diverse cultural backgrounds.

### Practical Implications

Integrative medical approaches, such as acupressure, show promise for promoting symptom management for BCS. Our results illustrate that integrative medicine is appealing to Black and Latina BCS and that remote delivery of the intervention through a video is acceptable. Clinicians and interventionists should attend to cultural adaptation for delivery of novel integrative care approaches. Our intervention is also attractive due to its low-cost, easy-to-disseminate format (video) that would be ideal for both community and clinical settings. Future dissemination and implementation work should examine how delivery of acupressure interventions could be facilitated in clinical or community settings, or through online referrals.

### Limitations and Future Directions

Our work is limited in generalizability by design since the acupressure video particularly focused on Black and Latina BCS in one geographic area. Other practitioners interested in acupressure should consider their target community and use processes such as learner verification and revision to modify instructional materials to best fit the population. Additionally, we specifically conducted the development and usability testing with the same participants to ensure that the video adaptations were accurately addressing their concerns and feedback. Future work with new samples of participants will increase generalizability of the video intervention to the wider population. Notably, none of the six BCS had indicated they practiced self-acupressure during the qualitative interviews. However, we did not assess participants’ past exposure or interest in acupressure at the time of study enrollment. It is possible that participants’ prior knowledge could influence responses. Future research may assess how individuals’ prior experience with acupressure influences their feedback and engagement in self-acupressure to inform intervention development and delivery.

Lastly, although our study demonstrated both high intervention acceptability and feasibility among the target population, it remains unclear whether a broader population of Black and Latina BCS would be satisfied with the current version. For example, Latinas have wide ethnic heritage; Latinas from Guatemala may interpret acupressure differently than Latinas from Puerto Rico. Black or African-American identifying women can also include American-born, Caribbean-born, or African-born populations in the USA. During the development of the self-acupressure video, we recognized that different cultural heritages might exist within the Latina and Black subgroups. Therefore, we consulted with community advocates for both Black and Latina BCS and gathered opinions from the six BCS through the qualitative interviews. Interestingly, we found that both Black and Latina participants shared a similar understanding of the acupressure techniques and had similar content- and format-related preferences (e.g., clarifying the difference between acupressure and acupuncture and adding Black and Latina women performing acupressure, testimonials, and colorful graphics and images). None of the participants in interviews suggested we should develop the video separately for Black and Latina BCS. The results guided our cultural adaptation of one single self-acupressure video.

Our work adapting the acupressure video may also be domain-specific and may not be applicable for all intervention topics, especially those involved in different levels of cultural acceptance across Black and Latina populations. This study suggests that developing intervention programs should consider the communities they work with and engage in early discussions with targeted community members and patients to make collaborative decisions about tailoring their interventions specifically to one or more cultural groups. Our next step is to test the video in a larger sample and evaluate its efficacy in symptom management for Black and Latina BCS through a randomized controlled trial. To establish the effectiveness of the self-acupressure intervention, future studies may examine facilitators and barriers to intervention implementation in clinical and community settings.

## Expected Impact and Conclusions

We are currently testing the intervention’s feasibility and efficacy with Black and Latina BCS in an online delivered self-acupressure intervention compared with usual care. In view of the current research on self-acupressure effects in reducing symptoms among cancer patients from other racial and ethnic groups, e.g.,[17], we expect that the current study will demonstrate favorable outcomes. If future work continues to demonstrate self-acupressure’s efficacy in improving symptom management, it has the potential to mitigate symptom management inequities and improve survivorship outcomes for Black and Latina BCS. Future work can consider intervention implementation, including integration into community clinics and group teach-ins on acupressure for specific populations.

## Supplementary Information

Below is the link to the electronic supplementary material.

Supplementary file1 (DOCX 672 KB)

## Data Availability

This study and its analysis plan were not formally pre-registered. De-identified data from this study are not available in a public archive. De-identified data from this study will be made available (as allowable according to institutional IRB standards) by emailing the corresponding author. There is not analytic code associated with this study. Materials used to conduct the study are not publically available.
