# Correction: Culturally Adapting a Video-Based Self-acupressure Intervention to Manage Symptoms for Black and Latina Breast Cancer Survivors

**DOI:** 10.1007/s13187-025-02760-6

**Published:** 2025-10-23

**Authors:** Katarina E. AuBuchon, Amrita Bonthu, Lourdes Inbar-Albo, Thelma D. Jones, Jacqueline Beale, Claudia Campos-Galván, Laura A. Logie, Carla Arieta, Geng-Hao Liu, Alejandra Hurtado-de-Mendoza, Suzanne C. Danhauer, Kristi D. Graves, Judy Huei-yu Wang

**Affiliations:** 1 Department of Oncology, Georgetown University, Washington, DC USA; 2 Department of Medicine, Case Western Reserve University School of Medicine, Cleveland, OH USA; 3 Thelma D. Jones Breast Cancer Fund, Washington, DC USA; 4 C2JB Consulting, Glenn Dale, MD USA; 5 The George Washington University Cancer Center, Washington, DC USA; 6 Nueva Vida, Inc, Alexandria, VA USA; 7 Inspire Integrative Medicine, Arnold, MD USA; 8 School of Traditional Chinese Medicine, Graduate Institute of Traditional Chinese Medicine, College of Medicine, Chang Gung University, Taoyuan, Taiwan; 9 Division of Acupuncture and Moxibustion, Center for Traditional Chinese Medicine, Linkou Chang Gung Memorial Hospital, Taoyuan, Taiwan; 10 Department of Social Sciences & Health Policy, Division of Public Health Sciences, Wake Forest University School of Medicine, Winston-Salem, NC USA

**Correction: Journal of Cancer Education**

**https://doi.org/10.1007/s13187-025-02684-1**

The article Culturally Adapting a Video-Based Self-acupressure Intervention to Manage Symptoms for Black and Latina Breast Cancer Survivors, written by Katarina E. AuBuchon, Amrita Bonthu, Lourdes Inbar-Albo, Thelma D. Jones, Jacqueline Beale, Claudia Campos-Galván, Laura A. Logie, Carla Arieta, Geng-Hao Liu, Alejandra Hurtado-de-Mendoza, Suzanne C. Danhauer, Kristi D. Graves and Judy Huei-yu Wang was originally published electronically on the publisher’s internet portal on 12 July 2025 without open access. With the author(s)’ decision to opt for Open Choice the copyright of the article changed on October 2025 to © The Author(s) 2025 and the article is forthwith distributed under a Creative Commons Attribution 4.0 International License, which permits use, sharing, adaptation, distribution and reproduction in any medium or format, as long as you give appropriate credit to the original author(s) and the source, provide a link to the Creative Commons license, and indicate if changes were made. The images or other third party material in this article are included in the article’s Creative Commons licensee, unless indicated otherwise in a credit line to the material. If material is not included in the article’s Creative Commons license and your intended use is not permitted by statutory regulation or exceeds the permitted use, you will need to obtain permission directly from the copyright holder. To view a copy of this license, visit http://creativecommons.org/licenses/by/4.0.

The original article has been corrected.

Open Access This article is licensed under a Creative Commons Attribution 4.0 International License, which permits use, sharing, adaptation, distribution and reproduction in any medium or format, as long as you give appropriate credit to the original author(s) and the source, provide a link to the Creative Commons licence, and indicate if changes were made. The images or other third party material in this article are included in the article's Creative Commons licence, unless indicated otherwise in a credit line to the material. If material is not included in the article's Creative Commons licence and your intended use is not permitted by statutory regulation or exceeds the permitted use, you will need to obtain permission directly from the copyright holder. To view a copy of this licence, visit http://creativecommons.org/licenses/by/4.0/.

