# Supplementary material for: Culturally Adapting a Video-Based Self-acupressure Intervention to Manage Symptoms for Black and Latina Breast Cancer Survivors
Source: J Cancer Educ. 2025 Jul 12;41(3):488–97. doi: 10.1007/s13187-025-02684-1 (PMC13222224; doi:10.1007/s13187-025-02684-1)
Supplement: Supplementary file 1 — Supplementary file1 (DOCX 672 KB) [file 13187_2025_2684_MOESM1_ESM.docx]

**Supplementary Materials**

Article name: Culturally Adapting a Video-Based Self-acupressure Intervention to Manage Symptoms for Black and Latina Breast Cancer Survivors

Journal name: *Journal of Cancer Education*

Authors: Katarina E. AuBuchon, Amrita Bonthu, Lourdes Inbar-Albo, Thelma D. Jones, Jacqueline Beale, Claudia Campos-Galván, Laura A. Logie, Carla Arieta, Geng-Hao Liu, Alejandra Hurtado-de-Mendoza, Suzanne C. Danhauer, Kristi D. Graves, & Judy Huei-yu Wang

Corresponding Author: Judy Wang, PhD

# **Table S1. Self-acupressure educational video**

| Acupressure Theory | |
| --- | --- |
| Acupressure vs. Acupuncture | - Acupressure: Uses thumbs or fingers, softer effect, self-administrable - Acupuncture: Uses needles, stronger effect |
| Benefits of Acupressure | - Improves immunity, blood vessels, lymph - Strengthens joints and nerves - Manages pain, anxiety, depression |
| Mechanism of Action | - Chi flows through meridians; blockages cause disease - Connection between brain and acupoints via hormone release (e.g., endorphins) |
| Location of Acupoints | |
| Locating and Pressing Acupoints | - LI4 (Hegu), ST36 (Zusanli), SP6 (Sanyinjiao) - Explanation of how to find each point and its benefits |
| Directions on How to Press Acupoints | |
| Preparation for Acupressure | - Cut and clean nails - Apply lotion - Sit in a relaxed position |
| Steps for Acupressure | - Pointing, Pressing, Kneading, Pushing |
| Duration and Sides | - Press both sides of the body for 1 minute each |
| De Qi Sensation | - Experience of soreness, tingling, swelling, aching - Intensity varies by individual |
| Safety Guidelines | - Stop if experiencing adverse effects (dizziness, cold sweating, nausea, vomiting, skin wounds) |

*Note.* The educational video focused on three primary points of interest.

**Figure S1. Acupressure points targeted for breast cancer survivors’ symptoms**

| **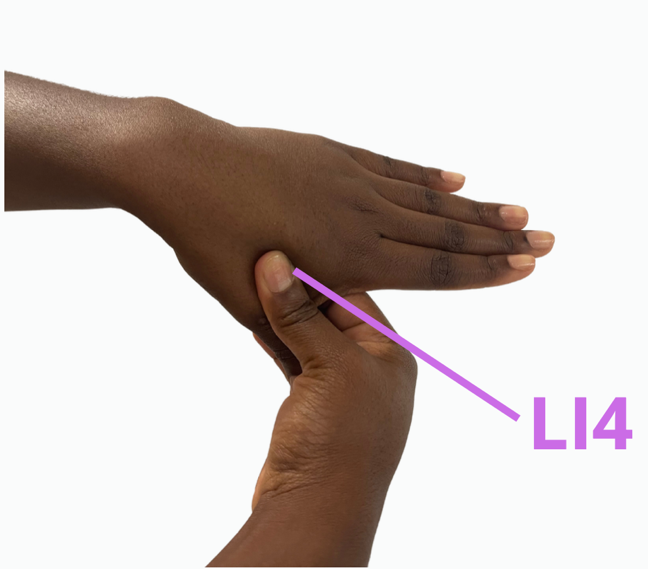** | |
| --- | --- |
| **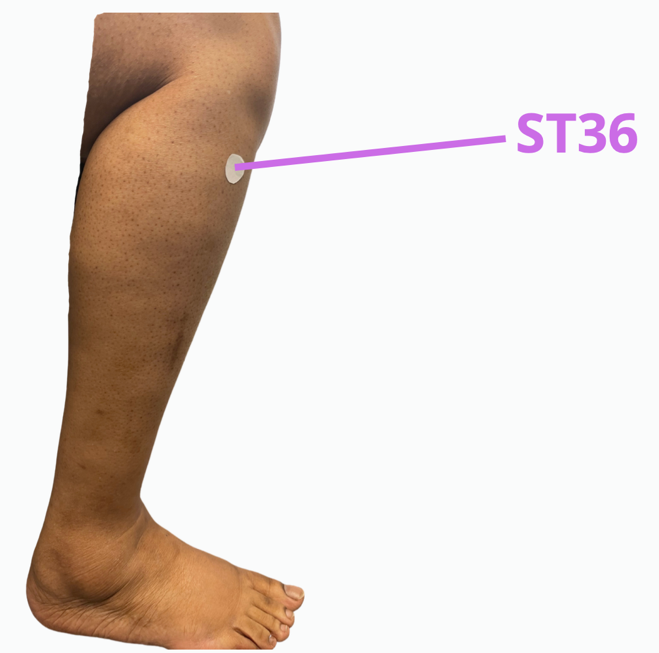** | **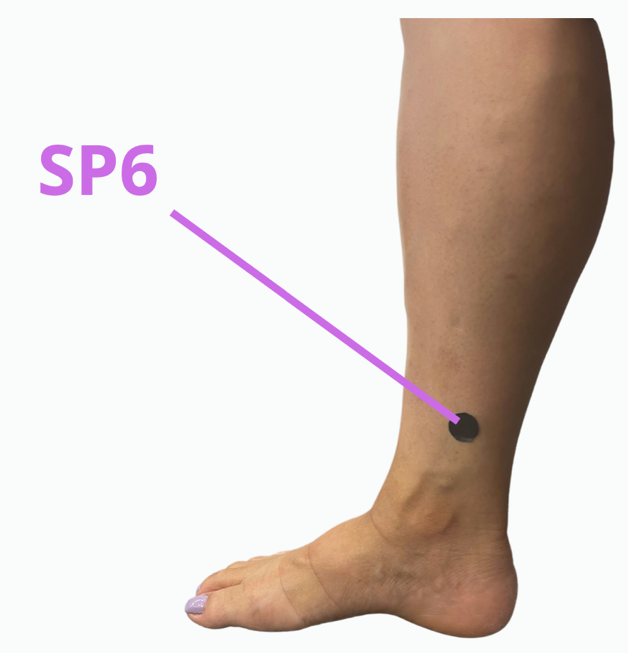** |

*Note.* Three acupressure points that are expected to influence BCS symptom management, used in the current study.
